# Proteoglycans Enhance the Therapeutic Effect of BMSC Transplantation on Osteoarthritis

**DOI:** 10.3390/bioengineering11111167

**Published:** 2024-11-20

**Authors:** Chunxiao Ran, Tianhao Liu, Yongming Bao, Weidan Wang, Dongling Xue, Guangxiao Yin, Xiuzhi Zhang, Dewei Zhao

**Affiliations:** 1 Department of Orthopedics, Affiliated Zhongshan Hospital of Dalian University, Dalian 116001, China; duke_19890903@126.com (C.R.); wangweidan@dlu.edu.cn (W.W.); dtysqdsh2000@gmail.com (D.X.); yinguangxiao1990@163.com (G.Y.); 2 Department of Life Science and Biotechnology, Dalian University of Technology, Dalian 116024, China; biosci@dlut.edu.cn

**Keywords:** proteoglycans, bone marrow mesenchymal stem cells, cartilage repair, paracrine, osteoarthritis

## Abstract

Background: The injection of bone mesenchymal stem cells (BMSCs) for osteoarthritis (OA) treatment fails to address the disrupted extracellular microenvironment, limiting the differentiation and paracrine functions of BMSCs and resulting in suboptimal therapeutic outcomes. Proteoglycans (PGs) promote cell differentiation, tissue repair, and microenvironment remodeling. This study investigated the potential of combining PGs with BMSCs to increase the efficacy of OA treatment. Methods: We evaluated the effects of PG on BMSC and chondrocyte functions by adding various PG concentrations to the culture media. Additionally, a Transwell system was used to assess the impact of PG on the communication between BMSCs and chondrocytes. The results of the in vitro experiment were verified by tissue staining and immunohistochemistry following the treatment of OA model rats. Results: Our findings indicate that PG effectively induces Col II expression in BMSCs and enhances the paracrine secretion of TGF-β1, thereby activating the TGF-β signaling pathway in chondrocytes and increasing PRG4 gene expression. Compared with the other groups, the BMSC/PG treatment group presented a smoother articular surface and more robust extracellular matrix than the other groups in vivo, with significantly increased expression and distribution of Smad2/3 and PRG4. Conclusions: PG enhances BMSC differentiation into chondrocytes and stimulates paracrine TGF-β1 secretion. Proteoglycans not only promote chondrocyte differentiation and paracrine TGF-β1 signaling in BMSCs but also increase the sensitivity of chondrocytes to TGF-β1 secreted from BMSCs, leading to PRG4 expression through the TGFR/Smad2/3 pathway. Proteoglycans can enhance the therapeutic effect of BMSC treatment on OA and have the potential to delay the degeneration of OA cartilage.

## 1. Introduction

Osteoarthritis (OA) is a common degenerative joint disease that progresses irreversibly. Globally, the number of OA patients has exceeded 300 million, and this figure continues to rise. OA not only severely impacts the quality of life of affected individuals but also places a significant burden on health care resources [1,2]. In recent years, numerous scholars have researched OA treatments, primarily focusing on novel drugs, drug delivery systems, and stem cell transplantation. The goal of these approaches is to improve OA symptoms by delivering sustained-release drugs or promoting cartilage repair through the chondrogenic differentiation of stem cells. However, these methods do not address the pathological mechanisms of OA or repair the abnormal tissue microenvironment within the joint, leading to suboptimal long-term outcomes [3,4].

In recent years, mesenchymal stem cells (MSCs) have garnered significant attention in the field of OA treatment. MSCs can self-renew and undergo multipotent differentiation, allowing them to differentiate into various cell lineages. In OA therapy, leveraging the chondrogenic differentiation and paracrine tissue repair properties of MSCs has shown promise in alleviating OA symptoms [5,6,7]. Although an intra-articular injection of MSCs can significantly inhibit the progression of cartilage damage in individuals with OA, the pathological OA microenvironment limits the differentiation efficacy and paracrine function of MSCs, resulting in their reduced capacity for cartilage regeneration and repair [5,8]. Therefore, an in-depth exploration of the tissue microenvironment in the context of OA and the optimization of MSC therapy through microenvironmental remodeling represent key research directions for addressing the challenges associated with OA treatment.

Proteoglycans (PGs), which are key components of the extracellular matrix (ECM) in cartilage, consist of glycosaminoglycan chains attached to a core protein and play crucial roles in constructing the microenvironment [9]. Pathological studies of OA have shown that insufficient ECM synthesis and increased degradation expose chondrocytes to significant external stress, exacerbating chondrocyte damage. The disruption of chondrocyte homeostasis further reduces PG synthesis [9,10,11]. In addition, PGs increase the expression of chondrogenic markers in MSCs by regulating various signaling factors, signal transduction, and cellular transport, thus promoting cartilage formation [9,11,12]. PGs can also bind to a variety of paracrine factors, modulating their bioavailability and release patterns, which are critical for the ability of MSCs to perform their tissue repair and immunomodulatory functions [13,14,15]. Therefore, PGs may be key factors in the reconstruction of the extracellular microenvironment of OA cartilage, providing a favorable niche for the differentiation, paracrine signaling, and immunoregulation of BMSCs and thereby facilitating the initiation of repair and regeneration in OA tissues.

This study systematically explores the impacts of PGs on BMSC differentiation and paracrine function through the reconstruction of the extracellular microenvironment and provides an in-depth analysis of the regulatory role of PGs in cell communication between BMSCs and chondrocytes. The results clearly demonstrate that the co-transplantation of BMSCs with PGs is highly effective in treating OA-induced cartilage damage, and the underlying mechanisms were elucidated. These findings provide a theoretical foundation for the application of BMSCs in combination with PGs in OA therapy.

## 2. Materials and Methods

### 2.1. Cell Culture

Chondrocytes (CM-R087, provided by the Shanghai Cell Bank, Shanghai, China) and BMSCs (ZQ0277, provided by ZQXZBIO, Shanghai, China) were grown in Dulbecco’s modified Eagle’s medium F12 (DMEM/F12) (Cytiva, Washington, DC, USA) supplemented with 100 µg/mL streptomycin, 100 IU/mL penicillin, and 10% fetal bovine serum (FBS; Gibco, Grand Island, NY, USA) in a 5% CO_2_ atmosphere and incubated at 37 °C. Cells at passages 3–6 were selected for experiments. Drawing on the study protocol for intra-articular injections of chondroitin sulfate (1–10 µg/100 µL) for the treatment of OA [16], we prepared DMEM/F12 containing PGs at concentrations of 5 µg/100 µL and 20 µg/100 µL.

### 2.2. Safranin O Staining of Cells

Chondrocytes and BMSCs were cultured in media supplemented with PGs (P5864, Sigma–Aldrich, Darmstadt, Germany) at concentrations of 5 µg/100 µL and 20 µg/100 µL, respectively, for 7 to 21 days. The cells were fixed with 4% paraformaldehyde. The cells were rinsed with deionized water, stained with Safranin O for 3 min, and then rinsed with deionized water after they were rinsed with 95% alcohol and anhydrous ethanol for 5 s to mix with the deionized water.

### 2.3. Real-Time Quantitative PCR Analysis of Chondrocytes and BMSCs

RNA was extracted and purified using an RNeasy Mini Kit (Qiagen, Germantown, MD, USA). The cDNA primers used were obtained from Dalian Ruizhong Biotechnology Co., Ltd. Dalian, China, and the RT-PCR analysis was subsequently performed. We normalized the cycle threshold (Ct) value of the gene of interest to the Ct value of GAPDH in the same sample and calculated the relative expression using the 2^−∆∆Ct^ method as follows:

Col II: F-GTCCTTCTGGAGATCAGGGTACT, R-ATTCCATTAGAGCCATCTTTGCC;
PRG4: F-GTGCCCATCAAAGCCTCTTATCA, R-CAGTGTTATCGCGGAAGTAACGA;
Sox 9: F-TGACCCGTTGATGTCCACTT, R-TCCACGAAGGGTCTCTTCTCG;
TGF-β1: F-CTGCTGACCCCCACTGATAC, R-TGACCCGTTGATGTCCACTT;
Smad2: F-TCCATCTTGCCATTCAC, R-TTCTTCCTGCCCATTCT.

### 2.4. Enzyme-Linked Immunosorbent Assay (ELISA)

BMSCs were inoculated into a 24-well plate with a density of 5 × 10^4^ cells per well for 7 days, divided into three groups (BMSC, BMSC PG 5 μg/100 μL, and BMSC PG 20 μg/100 μL). The protein concentration of TGF-β1 was measured using the ELISA method. Conditioned media from the coculture system were collected. According to the manufacturer’s instructions (Human Quantikine; R&D Systems, Minneapolis, MN, USA), the concentration of TGF-β1 in the medium was determined using the Human LAP (TGF-β1) Quantikine ELISA Kit. Three independent experiments were performed, and the measurements were taken using a Thermo Scientific (Thermo Scientific, Waltham, MA, USA) microplate reader.

### 2.5. Cell Counting Kit-8 Proliferation Assay

BMSCs or chondrocytes were seeded in a 96-well plate at a density of 5 × 10^3^ cells per well. The experiment was set up with four parallel groups: blank control (Blank), negative control (NC), PG 5 μg/100 μL, and PG 20 μg/100 μL, and measurements were taken at three time points: 24 h, 48 h, and 72 h. The cells were cultured in an incubator at 37 °C with 5% CO_2_. After 24 h, 48 h, and 72 h of culture, the medium was aspirated at each time point, washed twice with PBS, and replaced with fresh medium containing Cell Counting Kit-8 (CCK-8; Dojindo, Kumamoto, Japan) reagent. The cells were incubated for 2 h at 37 °C with 5% CO_2_, and absorbance was measured at 450 nm.

### 2.6. Cell Counting Protocol

Add the cell suspension to the 24-well plate at a density of 5 × 10^4^ (2 × 10^4^) cells per well and incubate for 24, 48, and 72 h (up to 7 days). Discard the culture medium, wash the cells with PBS, and add an appropriate amount of trypsin to digest the cells at 37 °C for 5 min, observing the cells as they round up and detach. Neutralize the trypsin with culture medium and gently pipette to fully disperse the cells, then transfer the cell suspension into a centrifuge tube. Centrifuge the cell suspension at 1000 rpm for 5 min and discard the supernatant. Resuspend the cells in an appropriate amount of culture medium to ensure an even distribution. Take 10 µL of the cell suspension and place it on the counting chamber for cell counting.

### 2.7. Transwell Coculture System

BMSCs and chondrocytes were cultured in a 6-well Transwell insert system (pore size 0.4 µm; Corning, NY, USA) to prevent direct cell-to-cell contact. Chondrocytes and BMSCs were seeded at a density of 5 × 10^4^ cells per well into the lower and upper chambers of the Transwell plates, respectively. As shown in figure, the cells on the left side of “/” are the cells in the upper chamber, and the cells on the right side are the cells in the lower chamber. All the cells were incubated at 37 °C with 5% CO_2_ in DMEM/F12 containing 10% FBS, with or without the addition of PGs (at a concentration of 5 µg/100 µL, P5864, Sigma–Aldrich, Darmstadt, Germany) and LY3200882 (10 µM, a TGF-β receptor inhibitor; MedChemExpress, Princeton, NJ, USA). The incubation period was 7 days, and the culture medium was replaced every three days.

### 2.8. Cellular Immunofluorescence Staining

The cells were washed with PBS and fixed with 4% paraformaldehyde for 15 min. After another wash, the cells were permeabilized with 0.2% Triton X-100 for 5 min and blocked for 1 h. The membranes were incubated with primary antibodies overnight at 4 °C. The primary antibodies were removed, and the cells were washed with PBS. A fluorescent secondary antibody was added, and the samples were incubated at room temperature in the dark for 40 min. DAPI was added, the samples were incubated in the dark for 5 min, and the slides were sealed. Green fluorescence was observed via fluorescence microscopy. Image-Pro Plus 6.0 software was used to objectively assess and determine the average integrated optical density (IOD) and optical density correction for all the photos. The total positive area and total integrated optical density of all images were quantified by the same criteria for the analysis of positive expression.

### 2.9. Establishment of the Animal OA Model and Intra-Articular Injection of Treatments

SPF Wistar male rats at 16–20 W were used (25 rats). The osteoarthritis model was established by removing the anterior and posterior cruciate ligaments and the medial meniscus. The medial longitudinal incision in the right knee joint was approximately 0.5 cm in length. The skin was cut, the subcutaneous tissue was separated, and the medial collateral ligament was exposed and cut. The knee joint cavity was opened, the anterior and posterior cruciate ligaments were exposed and cut, and the medial meniscus was exposed and removed. The bleeding was stopped thoroughly, the joint cavity was washed repeatedly with normal saline, the wound was sutured layer by layer, and penicillin ointment was applied to the wound to prevent infection. After one week, the experimental animals were divided into five groups: the normal group (5 unoperated rats), the OA group (5 rats), the PG group (5 rats), the BMSC group (5 rats), and the BMSC/PG group (5 rats).

For the PG treatment group, PGs were obtained from Sigma–Aldrich (Sigma–Aldrich, Darmstadt, Germany) and were dissolved in PBS to create 5 µg/100 µL PGs, which were injected at 100 µL into the knee joint cavity of OA model rats. The animals were injected biweekly and sacrificed after 4 weeks. For the BMSC group, a total of 2 × 10^6^ cells were injected into the knee joint cavity of OA model rats once every two weeks, and the rats were euthanized after 4 weeks. For the BMSC/PG group, PGs were dissolved in a suspension containing 2 × 10^6^ cells; 100 µL of the mixture of PGs and BMSC (5 µg/100 µL) was injected into the knee joint cavity of OA model rats once every two weeks, and the rats were sacrificed after 4 weeks.

### 2.10. Safranin O–Fast Green Staining of Paraffin Sections

The tissue sections were deparaffinized in water. The sections were incubated in a 0.1% safranin solution for 4 min and then rinsed 3 times with water. The sample was immersed in a 0.15% solid green dye solution for 4 min and then rinsed with running water for 1 min. The sections were washed with 1–2 min of a 1% glacial acetic acid solution and then rinsed with water for 1 min. The slices were dehydrated with a gradient of alcohol solutions and sealed with neutral gum.

### 2.11. Detection of Protein Levels Using Immunohistochemistry

The paraffin sections were air-dried at 60 °C for 2 h and routinely deparaffinized in water. The sections were incubated in a 3% hydrogen peroxide/methanol solution for 30 min at room temperature. After being washed with PBS, the sections were placed in the 0.01 M citrate buffer (pH = 6.0) working solution, and the antigens were retrieved under high pressure for 15 min. After the sections were washed with PBS, they were incubated with a 10% sheep serum blocking solution at room temperature for 1 h. The serum was removed, and the corresponding primary antibody was added to each section (BS1838/BS1231, Bioworld Technology, Co., Ltd., St Louis Park, MN, USA), which was incubated overnight at 4 °C in a humid chamber. The mixture was incubated at room temperature for 30 min. After washes with PBS, the corresponding secondary antibodies (sheep anti-mouse or anti-rabbit) were added (Proteintech Europe, Cat No: SA00004-2), and the cells were incubated at room temperature for 1 h. After the samples were rinsed with PBS, DAB working solution was added to control color development visualized under a microscope, and the color development was stopped with cold water. The slices were counterstained with hematoxylin, rapidly dehydrated with 95% and 100% alcohol, dried, and sealed with neutral gum.

Primary antibodies against the following proteins were used: MMP-13 (18165-1-AP, WUHAN SANYING, Wuhan, China); PRG4 (KHC0448, WUHAN SANYING, Wuhan, China); and Smad2/3 (BS1838, Bioword, Nanjing, China).

### 2.12. Statistical Analysis

Each experiment was repeated at least three times. Statistical analyses were performed using SPSS version 14.0 for Windows (SPSS, Inc., Chicago, IL, USA). We used Student’s *t* test for in vitro experiments. A *p* value of <0.05 was considered to indicate statistical significance. The letters a, b, c, and d indicate significant differences in *p* < 0.05 compared with those of the control group and the first, second, and third experimental groups, respectively.

## 3. Results

### 3.1. Chondrogenic Effect of Proteoglycans on Cartilage Cells

**Figure 1:**
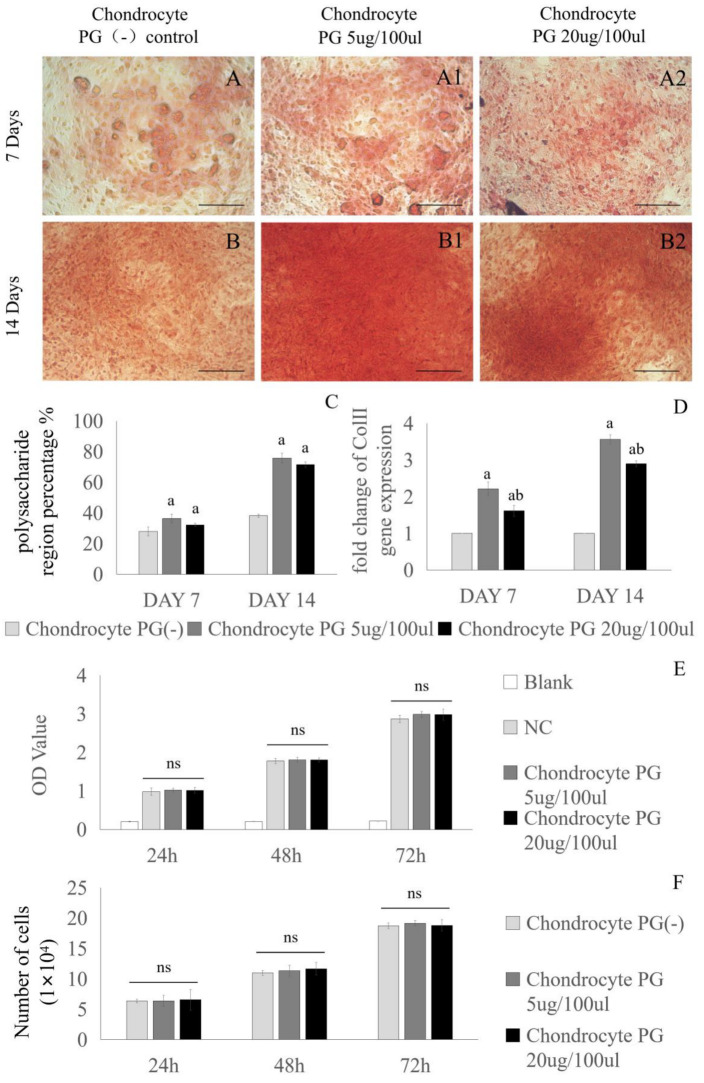
Effects of media supplemented with different concentrations of proteoglycans on chondrocyte activity. Chondrocytes were cultured with different concentrations of proteoglycans in medium (0 µg/100 µL, 5 µg/100 µL, or 20 µg/100 µL) for 7 days (**A**–**A2**) or 14 days (**B**–**B2**), after which the polysaccharide content in the extracellular matrix was observed by Safranin O staining. The polysaccharide-stained area in Panel (**A**–**B2**) was calculated, and the results are shown in Panel (**C**). Different concentrations of proteoglycans (0 µg/100 µL, 5 µg/100 µL, and 20 µg/100 µL) were added to cultured cartilage cells for 7 and 14 days, after which the expression of the Col II gene was measured via RT-PCR (**D**). The CCK-8 method was used to detect chondrocytes co-cultured with PG (5 µg/100 µL, 20 µg/100 µL) for 24 h, 48 h, and 72 h (**E**). Chondrocytes were co-cultured with PG (5 µg/100 µL, 20 µg/100 µL) for 24 h, 48 h, and 72 h (**F**). The scale bar in the figure represents 250 µm. Chon: chondrocyte. PG: proteoglycan. NC: negative control. a: indicates a significant difference in *p* < 0.05 compared with the Chon group. b: indicates a significant difference (*p* < 0.05) compared with the Chon + PG (5 µg/100 µL) group. ns: not significant.

Chondrocytes were cultured in media supplemented with different concentrations of PGs (0 µg/100 µL, 5 µg/100 µL, and 20 µg/100 µL) for 7 and 14 days to analyze the effect of PGs on chondroblast activity. The proteoglycan-producing ability of the chondrocytes was detected by Safranin O staining, and the expression of Col II in the chondrocytes was detected by PCR. When the chondrocytes were cultured for 7 days or 14 days, Safranin O staining revealed that the chondrocytes in the PG-supplemented medium secreted more extracellular matrix than did the chondrocytes in the PG-deficient medium, but a significant difference was not observed between the two PG concentrations (Figure 1A,B2). In terms of the percentage of polysaccharide-stained area, the area of extracellular matrix produced by chondrocytes was significantly increased following the addition of PGs. However, no statistically significant difference in the capacity of chondrocytes to synthesize polysaccharides was detected between the concentrations of 5 µg/100 µL and 20 µg/100 µL PGs (Figure 1C). The PCR results revealed that the level of Col II gene expression in chondrocytes in the 5 µg/100 µL PG and 20 µg/100 µL PG groups was significantly higher than that in the PG (-) group, and the Col II expression level in the 5 µg/100 µL PG group was significantly higher than that in the 20 µg/100 µL PG group (Figure 1D). The CCK-8 assay revealed that chondrocyte viability in both concentrations of PG-containing media exhibited no statistically significant difference compared to that observed in the basal medium (Figure 1E,F).

### 3.2. Effect of Proteoglycans on the Chondrogenic Differentiation of BMSCs

**Figure 2:**
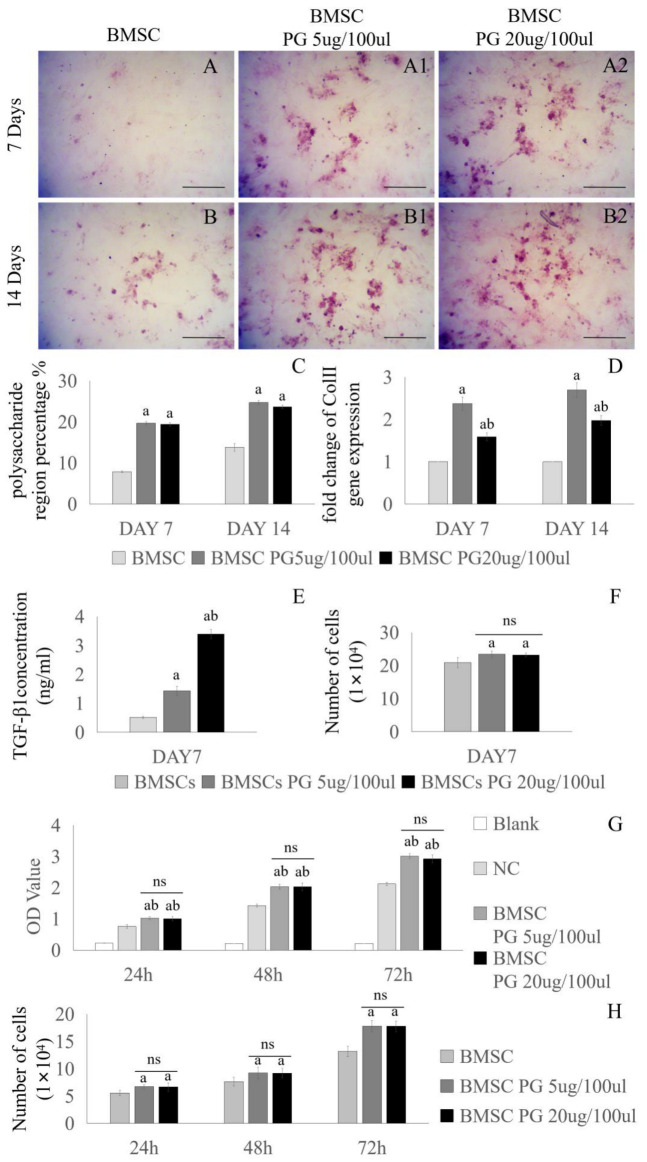
Effects of media supplemented with different concentrations of proteoglycans on BMSCs. Different concentrations of proteoglycans in medium (0 µg/100 µL, 5 µg/100 µL, or 20 µg/100 µL) were cultured with BMSCs for 7 days (**A**–**A2**) and 14 days (**B**–**B2**), and Safranin O staining was used to observe the content of the cartilage extracellular matrix. The polysaccharide-stained area in Panel (**A**–**B2**) was calculated, and the results are shown in Panel (**C**). BMSCs were cultured with different concentrations of proteoglycans in medium (0 µg/100 µL, 5 µg/100 µL, or 20 µg/100 µL) for 7 and 14 days, and the expression level of the Col II gene in the cells was detected by RT-PCR (**D**). BMSCs were cultured with basic medium or medium containing PG (5 µg/100 µL, 20 µg/100 µL) for 7 days, and the concentration of TGF-β1 was measured by ELISA (**E**). The three groups of cells were counted under the same conditions, and there was no statistical difference in the number of cells between the PG 5 µg/100 µL and PG 20 µg/100 µL groups (**F**). The CCK-8 method was used to detect BMSCs co-cultured with PG (5 µg/100 µL, 20 µg/100 µL) for 24 h, 48 h, and 72 h (**G**). BMSCs were co-cultured with PG (5 µg/100 µL, 20 µg/100 µL) for 24 h, 48 h, and 72 h (**H**). NC: negative control. The scale bar in (**A**–**B2**) is 250 µm. a: indicates a significant difference (*p* < 0.05) compared with the first group; b: indicates a significant difference (*p* < 0.05) compared with the second group; ns: not significant.

BMSCs were cultured in media supplemented with different concentrations of PGs (0 µg/100 µL, 5 µg/100 µL, or 20 µg/100 µL) for 7 or 14 days to analyze the effect of PGs on BMSC activity. The ability of BMSCs to produce proteoglycans was detected by Safranin O staining, and the expression of Col II in BMSCs was detected by PCR. The BMSCs were cultured for 7 days or 14 days. According to the statistical analysis of the area of polysaccharide staining, Safranin O staining revealed that BMSCs cultured in PG-supplemented medium secreted more extracellular matrix than chondrocytes cultured in PG-free medium did, but a significant difference was not observed between the 5 µg/100 µL PG group and the 20 µg/100 µL PG group (Figure 2A–C). The PCR results revealed that the expression level of the Col II gene in the BMSCs in the 5 µg/100 µL PG and 20 µg/100 µL PG groups was significantly higher than that in the PG(-) group. The expression level of the Col II gene in the 5 µg/100 µL PG group was significantly higher than that in the 20 µg/100 µL PG group at 7 days (Figure 2D). In addition, the content of TGF-β1 in the medium was detected by ELISA at day 7. It was found that the content of TGF-β1 secreted by BMSCs in PG medium of 5 µg/100 µL was 1.43 ± 0.15 ng/mL, while the content of TGF-β1 in PG medium of 20 µg/100 µL was 3.39 ± 0.16 ng/mL (Figure 2E). There was no significant difference in the number of cells in the three groups under the same conditions (Figure 2F). Studies have shown that low concentrations of TGF-β1 promote the differentiation of MSCs into chondrocytes and the expression of Col II, while high concentrations inhibit this differentiation [17,18]. The CCK-8 assay showed better cell viability of BMSCs in the two concentrations of PG-containing media compared to the base medium (Figure 2G,H). Given the effects of the two concentrations of PGs on the function of chondrocytes and BMSCs, we believe that the 5 µg/100 µL concentration of PGs is more suitable for the treatment of OA combined with the intra-articular injection of BMSCs.

### 3.3. PG Affects Intercellular Communication Between BMSCs and Chondrocytes Through the TGF-β Signaling Pathway

**Figure 3:**
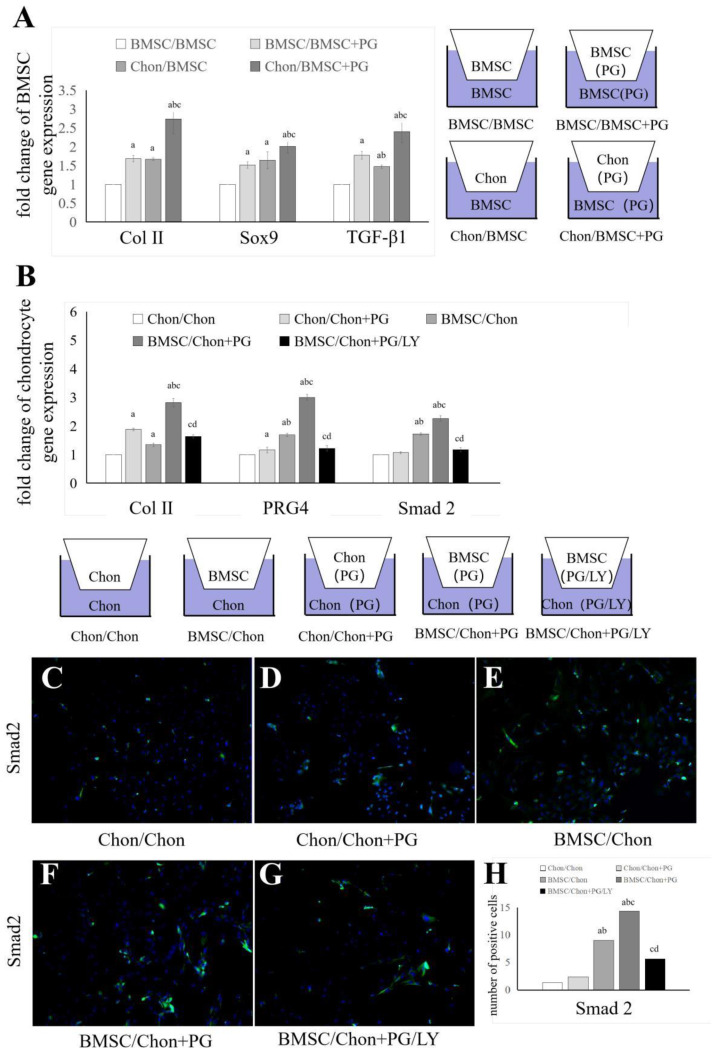
Effects of PGs on intercellular communication between BMSCs and chondrocytes in a Transwell system. The Transwell system was used to coculture chondrocytes and BMSCs in the presence or absence of PGs. BMSCs, chondrocytes, and PGs were cultured separately or together in the Transwell system for 7 days. The gene expression of Col II, Sox9, and TGF-β1 in BMSCs in the lower chamber was detected (**A**). Chondrocytes were cultured separately or together with BMSCs and PGs in a Transwell system for 7 days, after which cells were treated with LY3200882, and Col II, PRG 4, and Smad2 gene expression in the BMS/Chon + PG/LY group was detected by PCR (**B**). The Smad2/3 protein was detected by cellular immunofluorescence staining (**C**–**G**). (**H**) Shows the difference in the number of positive cells. Chon: chondrocytes, LY: LY3200882. The letters a, b, c, and d indicate significant differences (*p* < 0.05) compared with the 1st, 2nd, 3rd, and 4th groups.

We established a Transwell system and performed PCR on the cells in the lower chambers to explore the effect of PG on intercellular communication between BMSCs and chondrocytes. We established four coculture systems to elucidate the effects of chondrocytes on BMSCs in the presence or absence of PG (5 µg/100 µL) (Figure 3A). The expression levels of Col II, Sox9, and TGF-β1 in the lower chamber of the BMSCs were assessed using PCR. The results indicated that coculture with chondrocytes increased the expression of Col II, Sox9, and TGF-β1 in BMSCs, a phenomenon that was markedly enhanced in the presence of PGs. We further elucidated the differences in intercellular communication between BMSCs and chondrocytes in the presence or absence of PGs by establishing five coculture systems (Figure 3B). The expression levels of Col II, PRG4, and Smad2 in chondrocytes in the lower chamber were assessed using PCR. The results showed that coculture with BMSCs upregulated the expression of Col II, PRG4, and Smad2 in chondrocytes, a phenomenon that was significantly enhanced in the presence of PGs. This effect was reversed by the addition of the TGF-β receptor I inhibitor LY3200882. We performed Smad2 immunofluorescence staining on chondrocytes in the lower chamber based on the groups shown in Figure 3B to confirm that the upregulation of Smad2 in chondrocytes leads to the activation of Smad2 proteins. The results, which are illustrated in Figure 3C–H, revealed a trend consistent with the gene expression data.

### 3.4. BMSCs Combined with PGs Have a Better Protective Effect on the Articular Cartilage of Rats with OA

**Figure 4:**
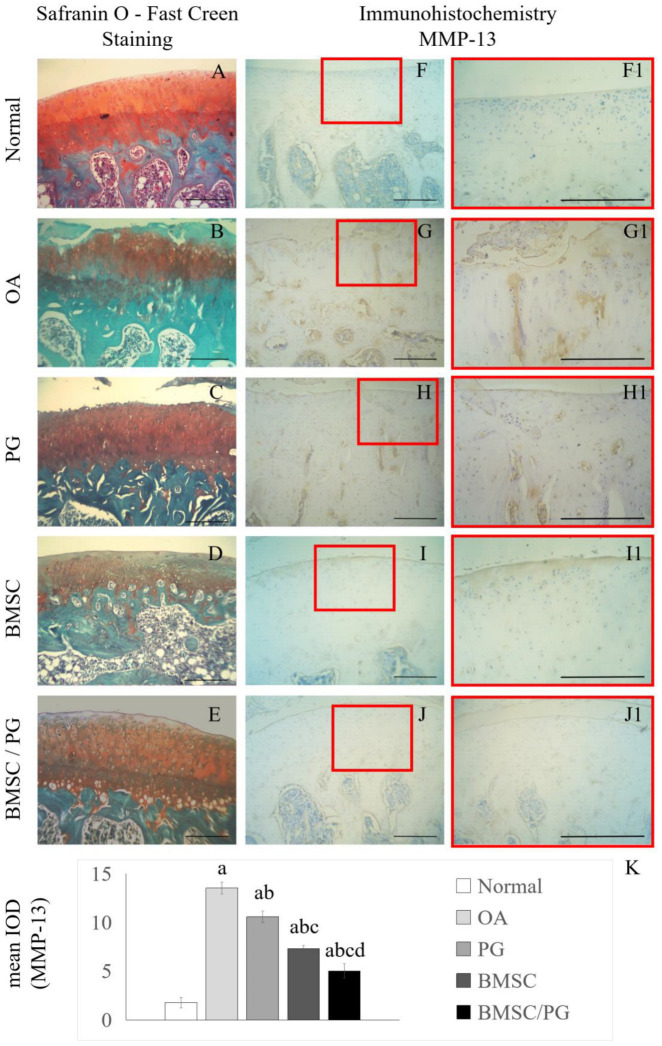
Intra-articular injection of BMSCs combined with PGs delays the degradation of articular cartilage in osteoarthritic joints. Safranin O–fast green staining was performed on paraffin sections from the normal group, OA group, PG group, BMSC group, and BMSC/PG group (**A**–**E**). IHC staining for MMP-13 was performed on paraffin sections from the normal group, OA group, PG group, BMSC group, and BMSC/PG group (**F**–**J**), and high-magnification images of the boxed areas in (**F**–**J**) are displayed on the right side (**F1**–**J1**). The mean IODs of the images in (**F1**–**J1**) are shown in (**K**). The scale bars in (**A**–**J1**) represent 250 µm. The letters a, b, c, and d indicate significant differences (*p* < 0.05) compared with the 1st, 2nd, 3rd, and 4th groups.

We performed experiments on animals in vivo to verify the effectiveness of PG and joint cartilage-derived BMSCs in treating OA. The OA rat model was established via the transection of the anterior cruciate ligament and removal of the medial meniscus. The OA model rats were divided into five groups (negative control group, OA control group, PG treatment group, BMSC treatment group, and BMSC/PG combined treatment group), and the proteoglycan concentration in the injected solution was 5 µg/100 µL. The experimental animals were sacrificed after 4 weeks. Paraffin-embedded tissue sections were stained. Safranin O staining clearly revealed that cartilage matrix staining was reduced and the articular cartilage surface was damaged in the OA group (Figure 4B); PG treatment restored cartilage matrix staining, but the surface of the joint was still severely damaged (Figure 4C). In the BMSC treatment group and the BMSC/PG treatment group, stronger staining of the cartilage matrix was observed, and the articular surface of the cartilage was flatter (Figure 4D,E). We investigated one of the main factors causing cartilage degeneration, MMP-13. Immunohistochemical staining revealed that the OA group had an extensive distribution of MMP-13 in full-thickness articular cartilage, and the PG treatment group had a slightly reduced distribution of MMP-13 (Figure 4G–H1). In the BMSC treatment group, MMP-13 was present mainly in the superficial layer of articular cartilage, and only a small amount of MMP-13 was distributed in the deep cartilage (Figure 4I,I1), whereas in the BMSC/PG treatment group, MMP-13 was sparsely distributed, and only a small amount of MMP-13 was produced in the deep cartilage (Figure 4J,J1).

### 3.5. BMSCs Combined with PGs Promoted the Production of PRG4 by Increasing the Level of Smad2 in Rat Chondrocytes

**Figure 5:**
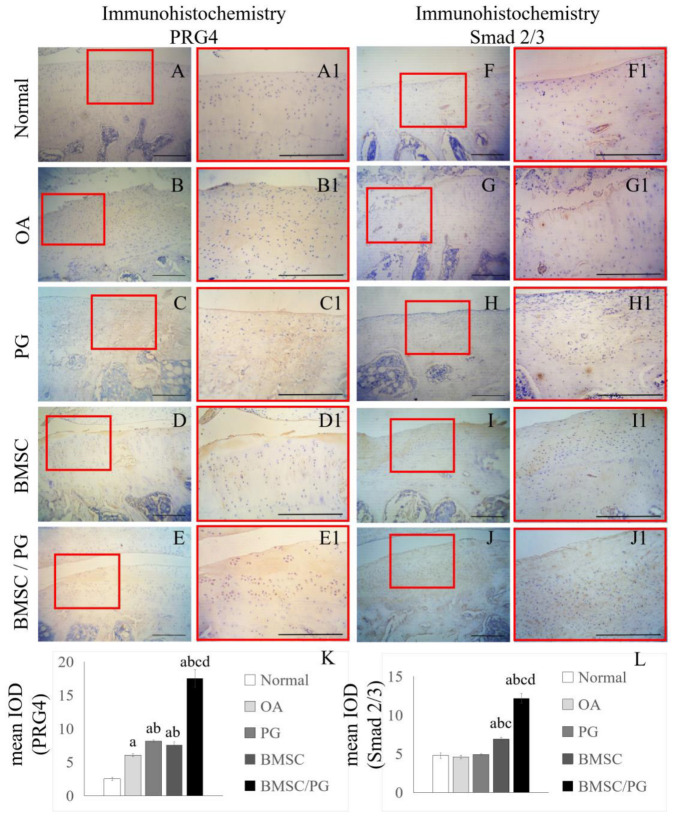
BMSCs combined with PGs promote the production of PRG4 in chondrocytes by upregulating the expression of Smad2/3. IHC staining for PRG4 was performed on paraffin sections from the normal group, OA group, PG group, BMSC group, and BMSC/PG group (**A**–**E**), and high-magnification images of the boxed areas in (**A**–**E**) are displayed on the right side (**A1**–**E1**). IHC staining for Smad2/3 was performed on paraffin sections from the normal group, OA group, PG group, BMSC group, and BMSC/PG group (**F**–**J**), and high-magnification images of the boxed areas in (**F**–**J**) are displayed on the right side (**F1**–**J1**). The mean IODs of the images in (**A1**–**E1**) and (**F1**–**J1**) are shown in (**K**,**L**). The scale bars in (**A**–**J1**) represent 250 µm. The letters a, b, c, and d indicate significant differences (*p* < 0.05) compared with the 1st, 2nd, 3rd, and 4th groups.

We performed immunohistochemical staining of paraffin sections from the five groups of OA model rats to determine the distribution of Smad2/3 and PRG4 in cartilage and verify the potential mechanism of BMSCs combined with PGs in the treatment of OA in vivo. We found that PRG4 was rarely detected in the articular cartilage of the control group (Figure 5A–B1). The content of PRG4 in the cartilage tissue of the PG treatment group and BMSC treatment group increased, and the distribution of PRG4 was concentrated in the superficial cartilage area (Figure 5C–D1). In the BMSC/PG treatment group, PRG4 covered the whole layer of cartilage tissue (Figure 5E,E1). We verified the finding from the in vitro experiment that the PRG4 concentration is related to Smad2/3 expression by comparing Smad2/3 immunohistochemical staining in the five groups of OA model rats. In the control group, little Smad2/3 antibody staining was observed in the articular cartilage (Figure 5F,F1). In the OA control group and PG treatment group, Smad2/3 staining was limited to the superficial cartilage area, and the staining in the PG treatment group was more obvious than that in the OA control group (Figure 5G–H1). Compared with that in the other three groups, Smad2/3 antibody staining was significantly increased in the BMSC treatment group and the BMSC/PG treatment group, and the distribution of Smad2/3 was spread throughout the whole cartilage layer. The degree of Smad2/3 antibody staining in the BMSC/PG treatment group was significantly greater than that in the BMSC treatment group (Figure 5I–J1).

## 4. Discussion

The pathogenesis of OA is complex, and the degradation of the extracellular matrix in cartilage is closely associated with abnormalities in PG synthesis and metabolism. PGs not only play a key role in regulating chondrocyte function but also improve the extracellular microenvironment, enhancing the differentiation potential and paracrine capacity of BMSCs to promote cartilage repair [19,20,21]. This study aimed to thoroughly investigate the mechanisms of PGs in OA treatment and evaluate their synergistic effects on reconstructing the extracellular microenvironment, promoting intercellular communication between BMSCs and chondrocytes, and facilitating cartilage repair.

In previous studies, chondroitin sulfate (CS) or monomeric proteoglycans have been utilized in intra-articular injections to treat OA. CS not only promotes cartilage extracellular matrix synthesis but also exerts anti-inflammatory and antioxidant effects, which enhance cartilage structure and alleviate joint pain by reducing oxidative stress and inflammation [22,23]. Chondroitin sulfate can induce MSC differentiation toward fibro-cartilage through interactions with cytokines, although its capacity to drive hyaline cartilage differentiation is limited [24]. Aggrecan, a key component of cartilage proteoglycans and a macromolecule within the proteoglycan family, has been investigated for its effects on OA progression. Krawetz et al. [25] showed that intra-articular injection of aggrecan into the knees of OA mice delayed cartilage degradation and OA progression. However, the OA pathological environment accelerates aggrecan degradation, which constrains its capacity for cartilage regeneration and repair.

Natural proteoglycans typically contain various types of glycosaminoglycans (e.g., heparan sulfate and chondroitin sulfate) and a core protein, structures that synergistically facilitate binding to multiple signaling molecules and growth factors essential for cartilage regeneration. In contrast, monomeric proteoglycans often lack this multifunctional binding capacity [9,26,27]. Moreover, the complex structure of natural proteoglycans renders them more resistant to degradation in vivo [28]. For instance, Syndecan-2 interacts with the pro-form of MMP-2 via its heparan sulfate (HS) chains, thereby inhibiting MMP-2 activation [29], while the core protein of versican forms complexes with MMP-9 domains to regulate MMP-9 activation modes [30]. Consequently, the structural stability of natural proteoglycans contributes to sustained cartilage repair outcomes. In this study, BMSC-combined PGs injection therapy is employed to construct a microenvironment that enhances intercellular signaling and modulates interactions between cells and the extracellular matrix. This approach aims to facilitate regenerative repair of articular cartilage by BMSCs, offering new insights and strategies for OA treatment.

Pathology studies of OA have shown that in the early stages of OA, the levels of PGs in the cartilage matrix increase in a compensatory manner. However, PG levels decrease due to proteolytic activity, ultimately leading to cartilage destruction [31]. Therefore, increasing PG levels may help delay cartilage degradation to some extent. In this study, PGs directly increased the activity of chondrocytes and promoted the increased production of ECM (Figure 1). Additionally, the expression of PRG4 in chondrocytes was upregulated in the presence of PGs (Chon/Chon + PG group) (Figure 3B). The long-term overexpression of PRG4 has been shown to prevent posttraumatic OA by inhibiting the transcriptional programs that promote cartilage catabolism and hypertrophy [32]. The production of the extracellular matrix and PRG4 creates favorable conditions for the repair of OA cartilage, indicating that PGs have the potential to reconstruct the extracellular microenvironment of OA cartilage.

BMSCs possess multipotent differentiation potential; however, controlling the direction of BMSC differentiation remains challenging. Our study revealed that culturing BMSCs with PGs derived from chondrocytes upregulated the expression of Col II and Sox9 while increasing the accumulation of extracellular PGs produced by BMSCs (Figure 2). Moreover, Sox9 expression in BMSCs was similarly upregulated in the BMSC/BMSC + PG group (Figure 3A), indicating that the PG-enriched environment promoted the differentiation of BMSCs into chondrocytes. Noncontact coculture of BMSCs with chondrocytes revealed that, under the influence of chondrocytes (Chon/BMSC group), the expression of Col II and Sox9 in BMSCs was upregulated. This effect was even more pronounced following the addition of PGs (Chon/BMSC + PG group) (Figure 3A), suggesting that PGs promote intercellular communication between BMSCs and chondrocytes. In in vivo experiments, we found that the transplantation of either BMSCs or PGs alone into the joint cavity of OA rats had a reparative effect on cartilage damage. However, the combined transplantation of BMSCs and PGs into the OA rat joint cavity had a significant synergistic effect, markedly increasing the cartilage matrix content and effectively restoring the integrity of the joint surface (Figure 4A–E). Hwang et al. studied the effects of morphogens derived from chondrocytes on MSC differentiation and reported that chondrocyte-secreted factors can promote the chondrogenesis of BMSCs [33]. Additionally, Sox9 overexpression has been shown to induce the accumulation of PGs and promote the differentiation of umbilical cord mesenchymal stem cells into chondrocytes without altering the MSC morphology [34]. These findings are consistent with our results, demonstrating that chondrocytes can promote BMSC chondrogenesis through paracrine factors. Furthermore, as a key component of the extracellular matrix in cartilage, PGs significantly increase the ability of BMSCs to differentiate into chondrocytes.

During the pathological process of OA, matrix metalloproteinases (MMPs) are synthesized in response to inflammatory stimuli and degrade key components of the cartilage matrix, such as collagen and PGs, leading to chondrocyte exposure to the external environment and exacerbating cartilage destruction. Among them, MMP-13 has a strong ability to degrade Col II and is a crucial marker of cartilage damage in individuals with OA [11]. Our previous research revealed that modulating the inflammatory environment is an important mechanism by which MSC transplantation can treat OA. MSCs achieve an immune balance by regulating the equilibrium between Treg and Th17 cells, as well as between M1 and M2 macrophages [5]. In this study, compared with the OA and PG groups, the BMSC group presented significantly suppressed synthesis of MMP-13 in chondrocytes because of the immunomodulatory effects of BMSCs (Figure 4I). Moreover, in the BMSC/PG group, the inhibition of MMP-13 synthesis in chondrocytes was further enhanced (Figure 4J), suggesting that the microenvironment constructed by PGs has a synergistic effect on augmenting the immunomodulatory function of BMSCs.

In our previous research, we reported that BMSCs promote intercellular communication between BMSCs and osteoblasts through the paracrine secretion of TGF-β1, thereby enhancing bone repair [35]. This finding highlights the critical role and mechanisms of BMSC paracrine signaling in OA treatment, where it exerts anti-inflammatory, immunomodulatory, and cell proliferation- and differentiation-promoting effects [5]. We used a Transwell system to exclude the effects of BMSC differentiation on the results and investigated the impact of BMSC paracrine signaling on chondrocytes in a PG-enriched environment. We found that BMSCs in the PG-enriched environment exhibited increased expression of TGF-β1 (BMSC/BMSC + PG group) (Figure 3A). The transplantation of BMSCs alone increased the expression of Col II, Smad2, and PRG4 in chondrocytes to a certain extent (BMSC/Chon group) (Figure 3B), whereas the combination of BMSCs and PGs had a synergistic effect (BMSC/Chon + PG group). This effect was abolished by the TGF-β1 receptor antagonist LY3200882 (BMSC/Chon + PG/LY group) (Figure 3B). Multiple studies have shown that TGF-β signaling can induce PRG4 expression in the superficial zone of articular cartilage through Smad2- or Smad3-mediated signaling pathways [36,37,38,39]. We further confirmed that TGF-β1 secreted by BMSCs can activate the synthesis of Smad2/3 in chondrocytes, thereby inducing PRG4 production and promoting the repair of OA cartilage. More importantly, the addition of PGs to create an extracellular microenvironment significantly enhanced intercellular communication between BMSCs and chondrocytes, further promoting OA cartilage repair. Consistent results were observed in our in vivo studies. Several studies have confirmed that the core proteoglycans within PGs can specifically bind to TGF-β1, significantly enhancing its ability to interact with cell surface receptors and increasing its biological activity [27,40]. Furthermore, PGs have been shown to regulate the activity, mobility, signal transduction, and intracellular transport of various signaling factors [41,42]. In studies of bone tissue, PGs were shown to regulate the gradient of TGF-β signaling by stabilizing TGF-β on the cell surface, leading to the enrichment of TGF-β in regions with increased proteoglycan levels [1,43,44]. Combining these findings with existing research, in addition to enhancing the paracrine secretion of TGF-β1 by BMSCs, PGs also play a role in facilitating the binding of TGF-β to its receptors on chondrocytes. The synergistic effects of PGs on these two processes promote cartilage regeneration through the TGFR-Smad2/3-PRG4 pathway.

**Figure 6:**
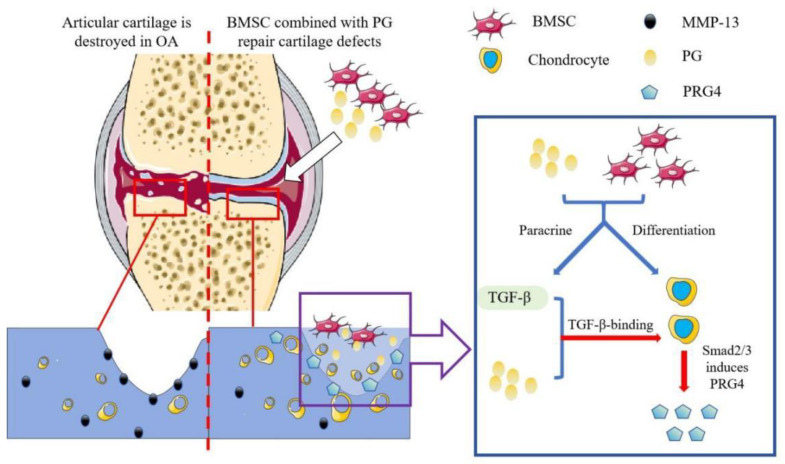
Mode of action of BMSCs combined with PG injection for OA treatment. BMSCs differentiate into chondrocytes under the influence of PGs. PGs enhance the paracrine effect of BMSCs on TGF-beta levels. The extracellular environment constructed by PGs increases the biological activity of TGF-β and stimulates chondrocytes to produce PRG4, thereby restoring the metabolic balance of chondrocytes and reducing the damage to articular cartilage caused by OA.

Proteoglycans are important extracellular matrix components in cartilage and play a key role in the construction of the microenvironment for OA treatment. Our findings suggest that proteoglycans not only promote chondrocyte differentiation and paracrine TGF-β1 signaling in BMSCs but also increase the sensitivity of chondrocytes to TGF-β1 secreted from BMSCs, leading to PRG4 expression through the TGFR/Smad2/3 pathway (Figure 6). Proteoglycans can enhance the therapeutic effect of BMSC treatment on OA.

## Data Availability

This paper presents all data required for the evaluation of its conclusions. Requests for further information related to this work can be directed to the corresponding author.
